# A combined approach of generalized additive model and bootstrap with small sample sets for fault diagnosis in fermentation process of glutamate

**DOI:** 10.1186/s12934-016-0528-1

**Published:** 2016-07-29

**Authors:** Chunbo Liu, Feng Pan, Yun Li

**Affiliations:** 1 Key Laboratory of Advanced Process Control for Light Industry, Ministry of Education, Jiangnan University, 1800 Lihu Avenue, Wuxi, 214122 Jiangsu China; 2 Mathematics, Informatics and Statistics Leeuwin Centre, Commonwealth Scientific and Industrial Research Organization (CSIRO), 65 Brockway Road, Floreat, WA 6014 Australia

**Keywords:** Fermentation process, Glutamate, Generalized additive model, Bootstrap, Small samples, Fault diagnosis

## Abstract

**Background:**

Glutamate is of great importance in food and pharmaceutical industries. There is still lack of effective statistical approaches for fault diagnosis in the fermentation process of glutamate. To date, the statistical approach based on generalized additive model (GAM) and bootstrap has not been used for fault diagnosis in fermentation processes, much less the fermentation process of glutamate with small samples sets.

**Results:**

A combined approach of GAM and bootstrap was developed for the online fault diagnosis in the fermentation process of glutamate with small sample sets. GAM was first used to model the relationship between glutamate production and different fermentation parameters using online data from four normal fermentation experiments of glutamate. The fitted GAM with fermentation time, dissolved oxygen, oxygen uptake rate and carbon dioxide evolution rate captured 99.6 % variance of glutamate production during fermentation process. Bootstrap was then used to quantify the uncertainty of the estimated production of glutamate from the fitted GAM using 95 % confidence interval. The proposed approach was then used for the online fault diagnosis in the abnormal fermentation processes of glutamate, and a fault was defined as the estimated production of glutamate fell outside the 95 % confidence interval. The online fault diagnosis based on the proposed approach identified not only the start of the fault in the fermentation process, but also the end of the fault when the fermentation conditions were back to normal. The proposed approach only used a small sample sets from normal fermentations excitements to establish the approach, and then only required online recorded data on fermentation parameters for fault diagnosis in the fermentation process of glutamate.

**Conclusions:**

The proposed approach based on GAM and bootstrap provides a new and effective way for the fault diagnosis in the fermentation process of glutamate with small sample sets.

## Background

Batch fermentation has been widely used in food, chemical and pharmaceutical industries to produce products of high value and low yield [1–4]. Online fault diagnosis of fermentation processes is of critical importance to ensure safe operation and stable yield of the final product. Even small faults on process parameters can decrease the quality and yield of final products. Early diagnosis of the behavior of abnormal process allows timely and corrective actions to be taken that not only can reduce the number of rejected batches, but also prevent the adverse effects on product quality and yield, and accidents [5, 6]. Fault diagnosis approaches in batch fermentation are needed to ensure the process and associated parameters within acceptable operation conditions [1, 7–9]. The dynamic behavior, strong nonlinearity, batch variations and multiplicity of operation phases make the fault diagnosis of the batch fermentation process very challenging [5, 10–13].

Multivariate statistical approaches such as multi-way principal component analysis (MPCA) and multi-way partial least-squares (MPLS) have been developed for fault diagnosis in batch fermentation processes [14–16]. But, the MPCA and MPLS methods have deficiency in solving problems with non-linear features [14–17]. These methods are based on the assumptions that the entire process data come from a single operation phase and the batch wise unfolded data follow a multivariate Gaussian distribution. Other statistical methods such as Kernel function based nonlinear PCA (KPCA), artificial neural networks (ANN) and support vector machine (SVM) have also been developed for fault diagnosis in fermentation processes [17–19]. These methods have the advantage to deal with fault problems in fermentation processes with nonlinear characteristics [20–22]. However, these methods are slow in fault detection in response to fault appearance and have random criteria for fault determination, which prevent their applications in fault diagnosis in fermentation processes [17]. In addition, these methods need substantial data to construct the model with a good performance for the fault diagnosis in fermentation process [23, 24], which are not suitable for small sample batch processes that cannot provide substantial training data. It is essential to further develop new and effective approaches for fault diagnosis in batch fermentation process.

Generalized additive model (GAM) is a statistical model for blending properties of generalized linear models with additive models [25–28]. GAM is a flexible and effective method for investigating non-linear relationships between the response and the set of explanatory variables with less restrictions in assumptions about the data distribution [29]. The model assumes that the dependent variables are dependent on the univariate smooth terms of independent variables rather than independent variables themselves [29]. GAM has been applied to investigate trends in water quality [30, 31], organic carbon content in soil [32] and factors affecting microcystin cellular quotas in the lake [29].

Bootstrap or bootstrap re-sampling was introduced as a computer-based method to calculate confidence intervals for parameters in circumstances where standard methods cannot be applied [33, 34]. It can draw a large number of re-sampled data from original data and it depends on fewer assumptions than classical statistical methods. Bootstrap can increase the robustness of fitted model in which a group of re-sampled data can be stochastically re-arranged to improve generalization capability of the fitted model [35–38]. Bootstrap methods are also an alternative for cross-validation in regression procedures when the number of observations is quite small and a validation set cannot be constructed from the original dataset [34, 39]. Bootstrap is very useful in solving problems that are too complicated for traditional statistical analysis [34]. Bootstrap has been used in signal-processing applications such as computer-aided diagnosis in breast ultrasound [34] and signal detection [37], spectral interval selection [39], and testing fundamental hypotheses in ecology [40].

Glutamate is widely used in food and pharmaceutical industries, with the production exceeds 2.2 million tons per year [41, 42]. However, there is still lack of effective statistical approaches for fault diagnosis in batch fermentation process of glutamate. A hybrid support vector machine and fuzzy reasoning based fault diagnosis system has been developed for glutamate fermentation, but this can only cluster the faults into three categories (shortage, medium and excess) based on initial biotin content variation [17]. To date, the approach based on GAM and bootstrap has not been used for the fault diagnosis in fermentation processes, much less the fermentation process of glutamate with small samples. In previous work, we successfully applied the GAM method to optimize the fermentation process of glutamate with improved production of glutamate [43]. In this study, a combined approach of GAM and bootstrap was developed for the online fault diagnosis in the fermentation process of glutamate with small sample sets. GAM was first used to model the relationship between glutamate production and different fermentation parameters using data from normal fermentation experiments of glutamate. The fitted GAM with fermentation time (T), dissolved oxygen (DO), oxygen uptake rate (OUR) and carbon dioxide evolution rate (CER) captured 99.6 % variance of glutamate production during fermentation process. Bootstrap re-sampling was then used to quantify the uncertainty of the estimated production of glutamate from the fitted GAM using 95 % confidence interval. The proposed approach based on GAM and bootstrap was used for the online fault diagnosis in the abnormal fermentation processes of glutamate, and a fault was defined as the estimated production of glutamate fell outside the 95 % confidence interval.

## Results and discussion

### Model construction

**Fig. 1:**
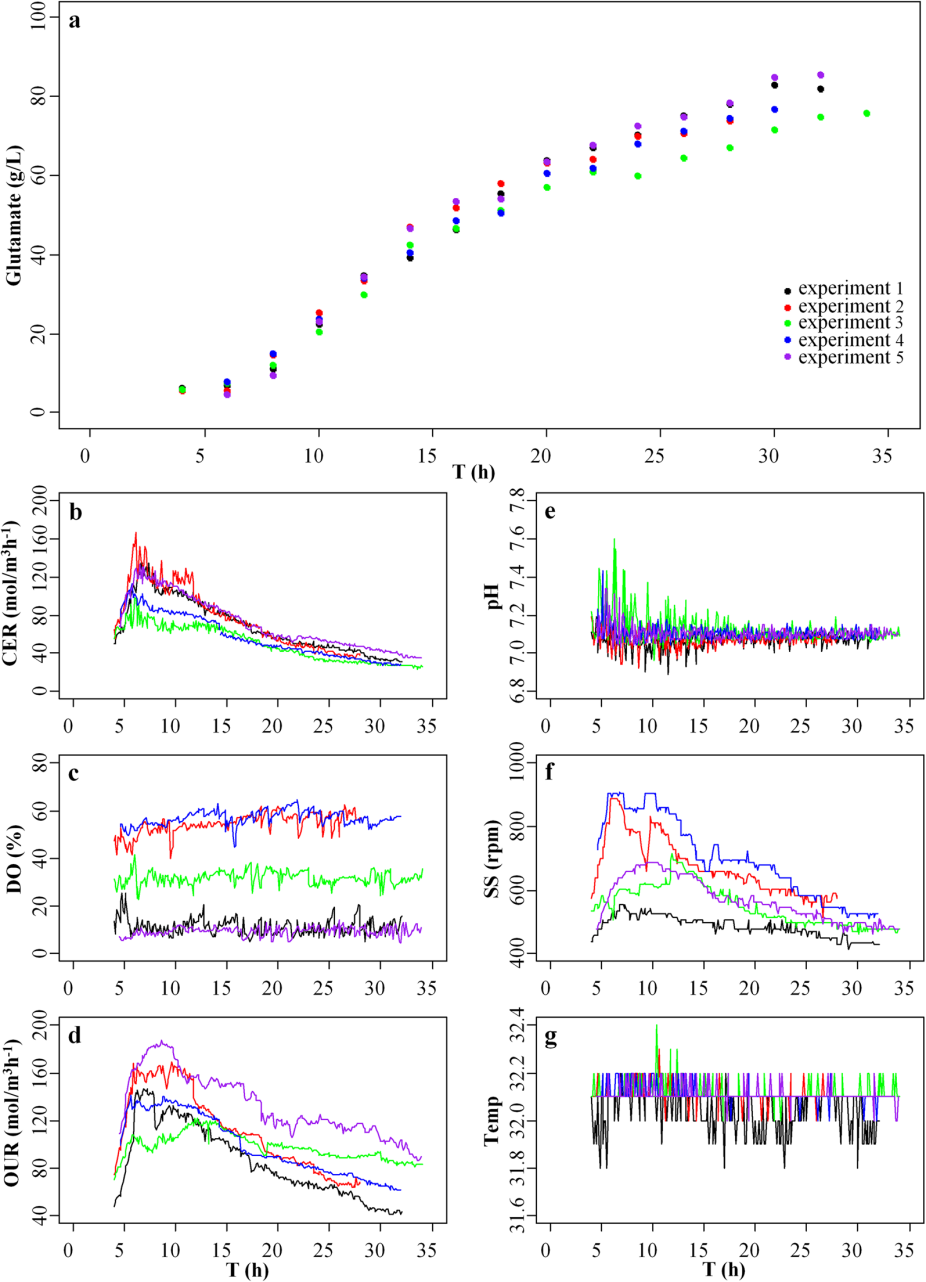
Data from five normal fermentation experiments of glutamate. **a** the offline data on glutamate production that were measured every 2 h during the fermentation process; the online data on (**b**) carbon dioxide evolution rate (CER), **c** dissolved oxygen (DO), **d** oxygen uptake rate (OUR), **e** pH, **f** stirring speed (SS) and **g** temperature (Temp) that were recorded every 6 min during the fermentation process

The offline data on glutamate production and the online data on different fermentation parameters for model construction and validation were collected from five normal fermentation experiments of glutamate (Fig. 1). In the normal fermentation experiments, the production of glutamate increased in a non-linear way during the fermentation process with the final production of glutamate between ~75 and ~85 g/L (Fig. 1a). The levels of CER increased from ~50 to ~170 mol/m^3^ h^−1^ during the early period from 4 to 7 h, and then dropped to ~40 mol/m^3^ h^−1^ (Fig. 1b). The levels of DO of the five normal experiments were between ~10 and ~55 % (Fig. 1c). The changing trend of OUR during the formation period was similar to that of CER (Fig. 1d), which confirmed the previous observation that there was a strong link between OUR and CER during the fermentation process of glutamate [24]. The pH of the five normal experiments was ~7.1 (Fig. 1e), the stirring speed was between 400 and 900 rpm (Fig. 1f), and the temperature was between 31.8 and 32.4 °C (Fig. 1g) during the fermentation period.

The training data from four randomly selected experiments were used to construct GAM and GLM. The fitted GAM showed a GCV score of 4 and an adjusted *R*^2^ of 0.996 while the fitted GLM showed a GCV score of 44 and an adjusted *R*^2^ of 0.940 (Table 1). This indicates that GAM was better than GLM in modeling the relationship between glutamate production and different fermentation parameters. The fitted GAM was defined as:

**Table 1:** The generalized linear model and generalized additive model constructed by training data

	Generalized linear model	Generalized additive model
Estimates for parametric functions		
Intercept	1466* (573)	47.35*** (0.22)
T	2.64*** (0.19)	–
DO	0.02 (0.08)	–
OUR	−0.01 (0.07)	–
CER	−0.06* (0.09)	–
SS	0.01 (0.02)	
pH	−3.01 (23.69)	–
Temp	−45.16* (17.70)	
Degrees of freedom for smooth terms		
s(T)	–	7.96***
s(DO)	–	2.34**
s(OUR)	–	3.00**
s(CER)	–	3.71***
Adjusted *R* ^2^	0.940	0.996
GCV score	44	4

Data in parentheses represent standard errors of the parametric functions

*T* fermentation time, *DO* dissolved oxygen, *OUR* oxygen uptake rate, *CER* carbon dioxide evolution rate, *SS* stirring speed, *Temp* temperature, *GCV* generalized cross-validation

* *P* < 0.05

** *P* < 0.01

*** *P* < 0.001

1 $$Glutamate = 4 7. 3 5+ s(T,7.96) + s(DO,2.34) + s(OUR,3.00) + s(CER,3.71)$$

And, the fitted model defined by Eq. (1) can capture 99.6 % variance of glutamate production. The performance of the fitted model was not significantly (*P* > 0.05) enhanced by including the remaining three fermentation parameters stirring speed, pH and temperature. This suggests that the production of glutamate was mainly attributed to the smooth functions of the four fermentation parameters T, DO, OUR and CER when GAM approach was used to model the relationship. And thus, the fitted GAM with the four significant factors T, DO, OUR and CER was used to estimate the production of glutamate for online fault diagnosis.

**Fig. 2:**
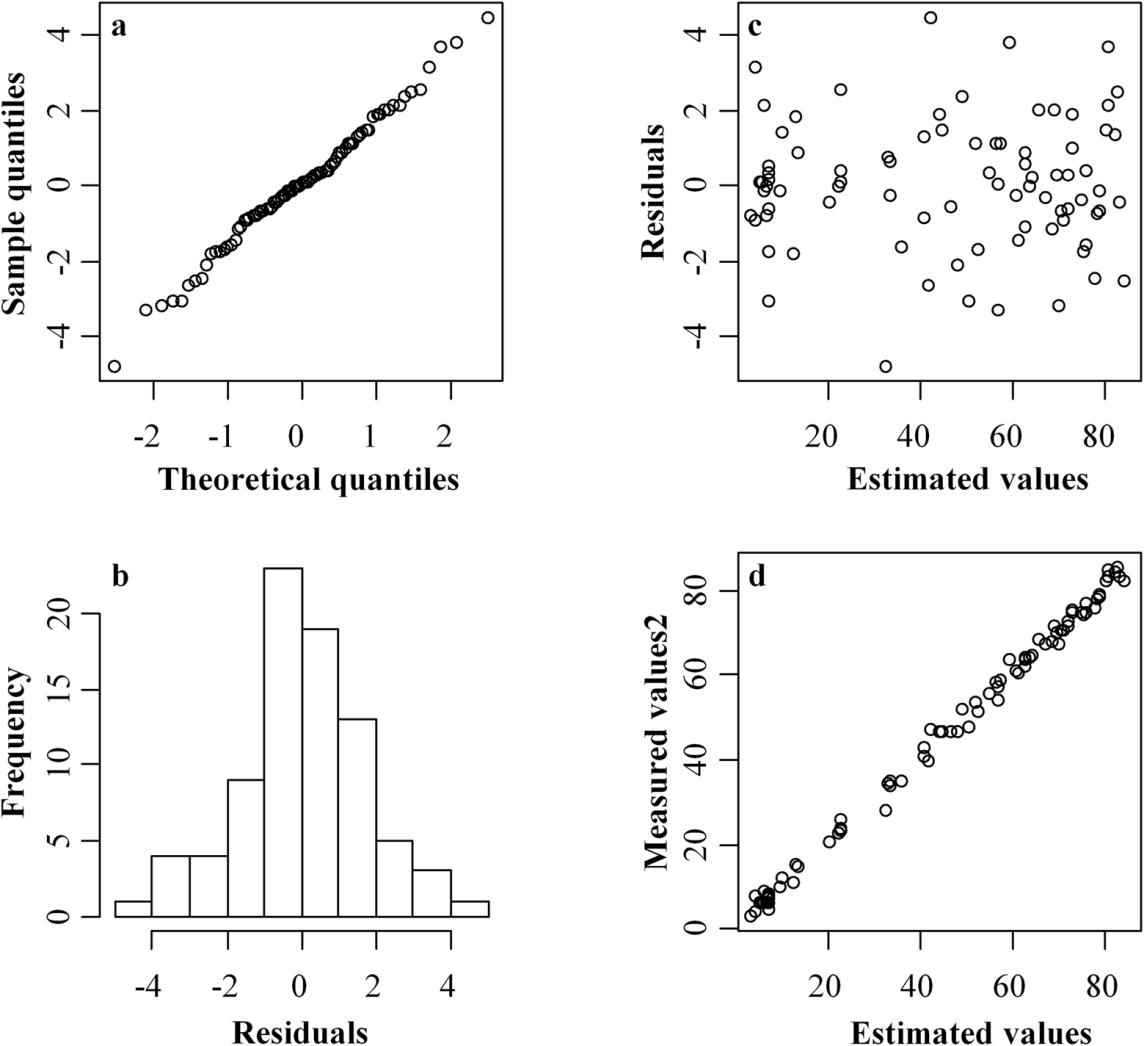
Diagnosis of the fitted generalized additive model. **a** normal Q–Q plot; **b** histogram of residuals; **c** residuals versus estimated values; **d** measured versus estimated values on glutamate production

Following diagnosis was conducted to check the validity of the fitted GAM defined by Eq. (1). The sampled data and residuals generated by the fitted GAM were close to normal distribution (Fig. 2a, b), suggesting the model followed the assumption required by Eq. (2). The residuals appeared as random scatters around zero without particular trend and pattern (Fig. 2c). This indicates there were no system errors due to the fitted GAM and the capability of the model to describe the effect of different parameters on the production of glutamate. There were no obvious influential outliers between estimated and measured values of glutamate production (Fig. 2d). The performance of the fitted GAM was also confirmed by the testing data. The measured values and estimated values on glutamate production from the fitted GAM using testing data was significantly correlated (*P* < 0.01), with a correlation coefficient of 0.996 and a root mean square error of 4.16 g/L.

### Bootstrap re-sample and confidence interval for glutamate production

**Fig. 3:**
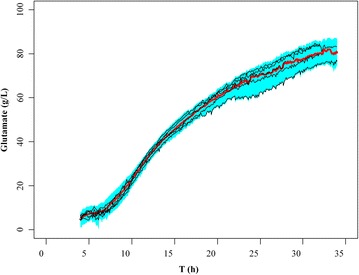
The 95 % confidence interval for glutamate production during fermentation process. The 95 % confidence interval (*shaded in green*) and mean values (*red curve*) for glutamate production were estimated from 1000 generalized additive models (GAMs) built by bootstrap re-sampling with replacement from the training data on glutamate production and fermentation parameters. *Black curves* represent the estimated glutamate production for the five normal fermentation processes from the fitted GAM built by the training data using the online recorded data on fermentation parameters

The fitted GAM was used to estimate glutamate production during fermentation process using online recorded data of the four fermentation parameters (T, CER, DO and OUR) from five normal fermentation experiments. The uncertainty of the estimated glutamate production was then quantified using 95 % confidence interval, which were estimated from 1000 GAMs built by bootstrap re-sampling with replacement from the training data on glutamate production and fermentation parameters (Fig. 3). It was evident that the estimated glutamate production from the fitted GAM using online recorded data of fermentation parameters from the five normal fermentation experiments all fell within the 95 % confidence interval for glutamate production; in addition, the means for glutamate production during fermentation process that were estimated from 1000 GAMs built by bootstrap re-sampling with replacement from the training data on glutamate production and fermentation parameters were within the estimated glutamate production from the five normal fermentation experiments. Therefore, online fault diagnosis in the fermentation process of glutamate was established by defining a fault when the estimated glutamate production from the fitted GAM fell outside the 95 % confidence interval using online recorded data of the four fermentation parameters (T, CER, DO and OUR) during the fermentation process.

### Fault diagnosis during fermentation process

Based on the 95 % confidence interval for glutamate production, when there is abnormal during fermentation process, the estimated production of glutamate from the fitted GAM using online recorded data of the fermentation parameters will fall outside the 95 % confidence interval, and an alarm to check the abnormal parameters can be issued immediately to avoid the decrease in the quality and production of glutamate due to fault accumulation. To demonstrate this, the fault diagnosis was conducted on two abnormal fermentation experiments of glutamate.

**Fig. 4:**
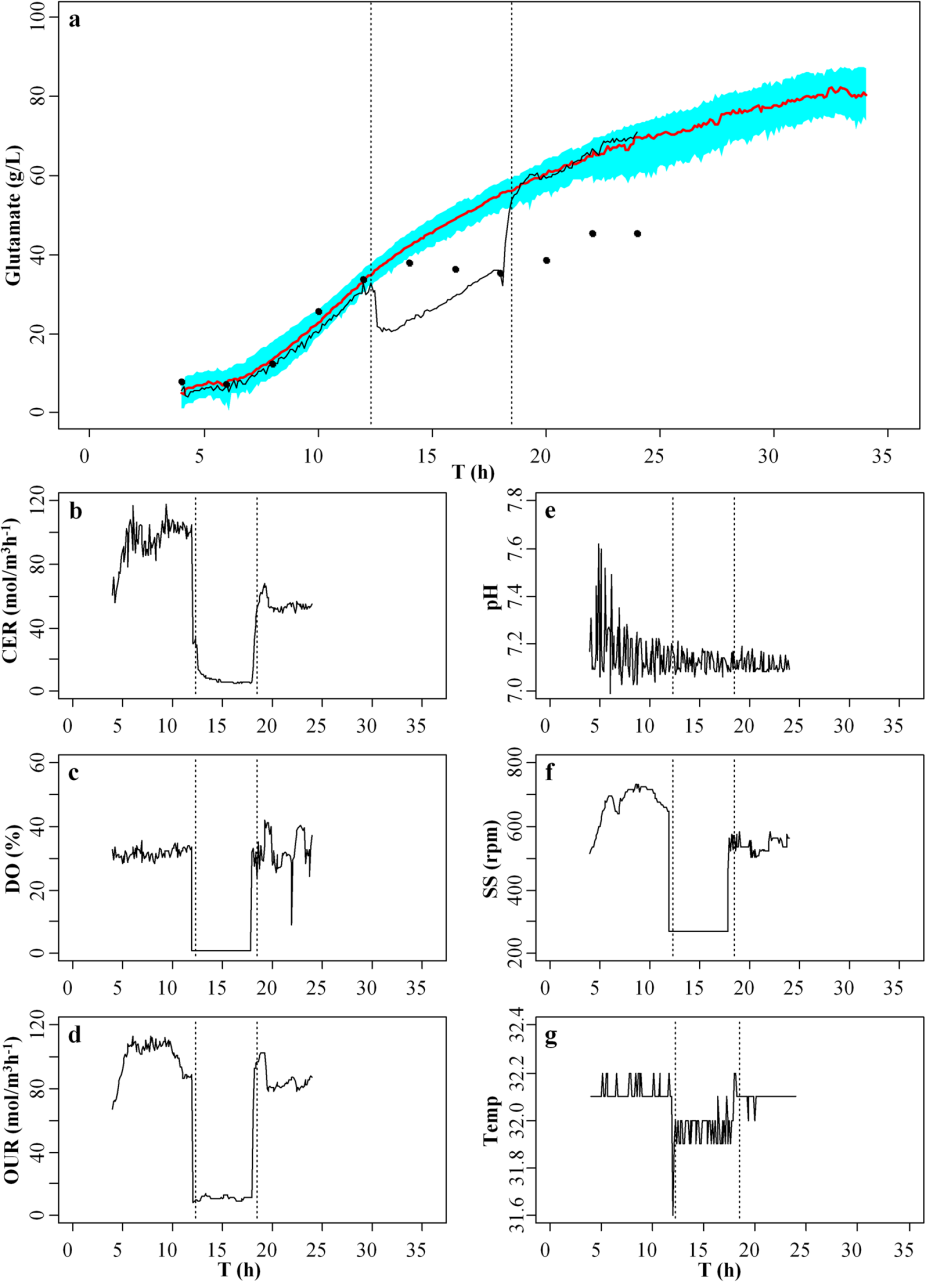
Fault diagnosis in the abnormal fermentation process of glutamate with fault source from stirring speed. **a** the 95 % confidence interval (*shaded in green*) and mean values (*red curve*) for glutamate production. The *black curve* represents the estimated production of glutamate from the fitted GAM using the online recorded data on fermentation parameters from this abnormal experiment. The *black dots* represent the offline measured production of glutamate from this abnormal experiment. **b**–**g** online recorded data on the fermentation parameter (**b**) carbon dioxide evolution rate (CER), **c** dissolved oxygen (DO), **d** oxygen uptake rate (OUR), **e** pH, **f** stirring speed (SS) and **g** temperature (Temp) from this abnormal experiment

The fault diagnosis was firstly conducted on the abnormal fermentation experiments of glutamate with the fault source from stirring speed (Fig. 4). It was shown that the estimated glutamate production from the fitted GAM using the online recorded data of T, CER, DO and OUR from this experiment fell outside of the 95 % confidence interval during the fermentation period from 12.3 to 18.5 h (Fig. 4a). Through the investigation on the online recorded data of different fermentation parameters, it was found that CER and OUR both fell below the level 20 mol/m^3^ h^−1^ during the same period (Fig. 4b, d), and the level of DO was nearly close to zero (Fig. 4c). There was a sudden drop of stirring speed to below 300 rpm during this period (Fig. 4f), and the abnormal stirring speed resulted in the very low levels of CER, DO and OUR during the same period, which could induce severe oxygen depletion to *Corynebacterium Glutamicum*. The actual fault in this experiment confirmed that the stirring speed of the fermenter started abnormal at about 12.3 h, and the fault was removed at about 18.5 h. After 18.5 h, the levels of stirring speed, CER, DO and OUR were back to normal and the estimated glutamate production returned back to the 95 % confidence interval (Fig. 4a).

**Fig. 5:**
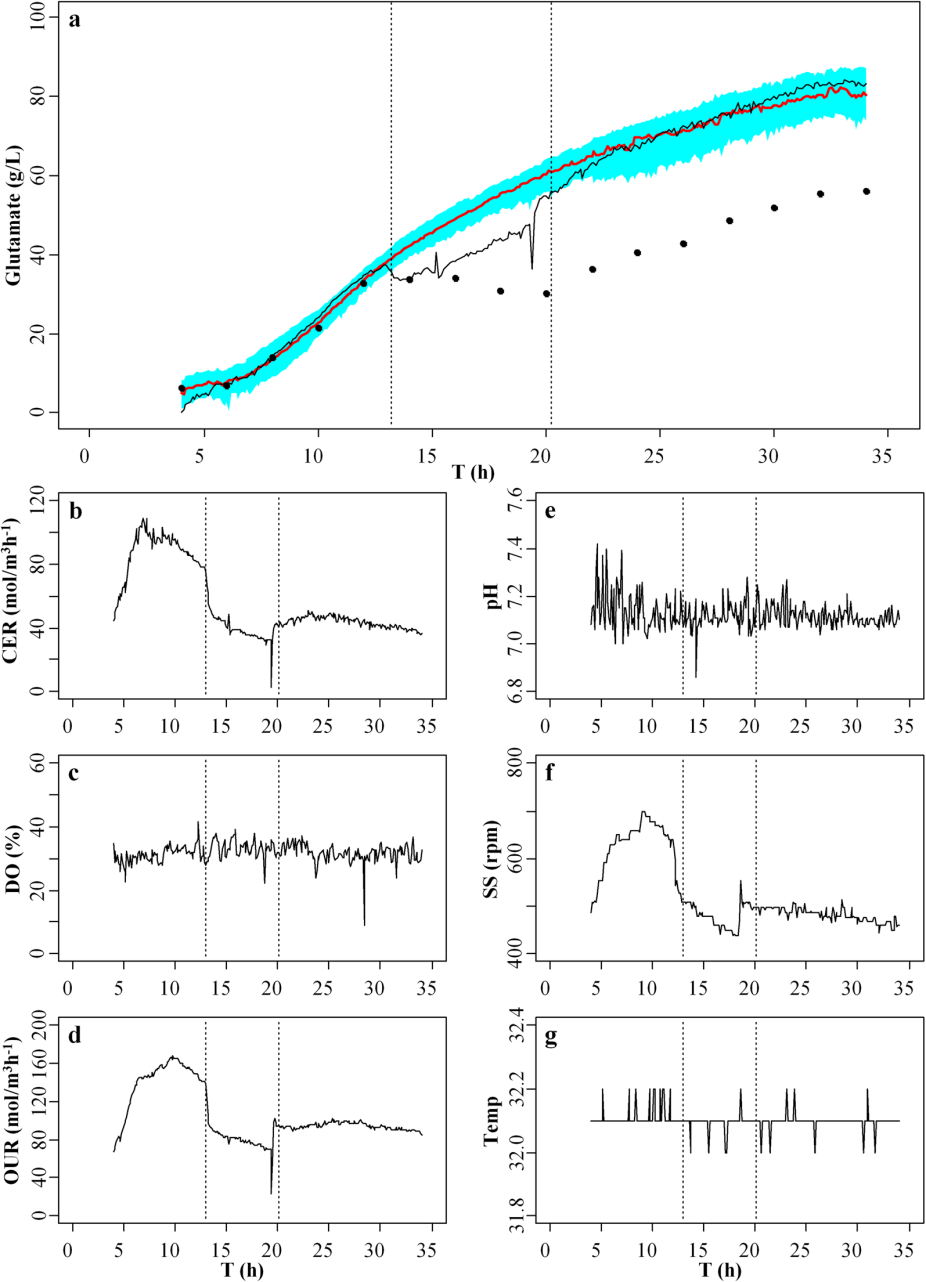
Fault diagnosis in the abnormal fermentation of glutamate with fault source from the human operation mistake. The mistake was due to NaOH solution was used instead of ammonia water to maintain the pH level during the operation. **a** the 95 % confidence interval (*shaded in green*) and mean values (*red curve*) for glutamate production. The *black curve* represents the estimated production of glutamate from the fitted GAM using the online recorded data on fermentation parameters from this abnormal experiment. The *black dots* represent the offline measured production of glutamate from this abnormal experiment. **b**–**g** online recorded data on the fermentation parameter (**b**) carbon dioxide evolution rate (CER), **c** dissolved oxygen (DO), **d** oxygen uptake rate (OUR), **e** pH, **f** stirring speed (SS) and **g** temperature (Temp) from this abnormal experiment

The fault diagnosis was also conducted on another abnormal fermentation experiment of glutamate with the fault source from the human operation mistake that NaOH solution was used instead of ammonia water to maintain the pH level during fermentation (Fig. 5). It was shown that the estimated glutamate production by the fitted GAM using the online data of T, CER, DO and OUR from this experiment fell outside the 95 % confidence interval for glutamate production during the period from 13 to 20 h (Fig. 5a). By checking the online recoded data on different fermentation parameters, it was found that there was a drop of CER and OUR during the period from 13 to 19 h, and a drop of stirring speed from 13 to 18 h while the other fermentation parameters were maintained at normal conditions (Fig. 5b–g). However, the stirring speed was within the normal range of 400–900 rpm during the period from 13 to 20 h; this indicated that the changes of stirring speed in this experiment was not attributed to the abnormal of OUR and CER. As the stirring speed, DO and temperature were all normal in this experiment, pH was the parameter need to be further checked so as to find the possible fault source because the level of pH could be still maintained at a normal range under certain abnormal conditions. After checking, an operation mistake was found that NaOH solution was used instead of ammonia water to maintain the pH level during fermentation. Such fault was very difficult to be identified by human eyes as the level of pH was still maintained at a normal range when ammonia water was replaced by NaOH solution during the operation. But, NaOH solution was harmful to the growth of *C. Glutamicum* and it cannot serve as nitrogen source required by glutamate synthesis during the fermentation process as provided by the added ammonia water [24].

Although the fault source from the operation mistake, which NaOH solution was used instead of ammonia water, was not easy to be identified in this experiment by artificial check of different fermentation parameters, the abnormal condition was still detected by the proposed approach with the estimated glutamate production fell outside its 95 % confidence interval. The start time of the fault was identified at 13 h when the estimated glutamate production fell outside the 95 % confidence interval, and the end time of fault was identified at 20 h as after this the estimated glutamate production returned back to the 95 % confidence interval (Fig. 5a). After the fault was removed at 20 h, the final production of glutamate was 56.1 g/L at the end of this experiment. These results suggest that if the fault source can be identified and removed timely during the fermentation process, the final production of glutamate may be still maintained at a satisfied level, although it was lower than the final production from normal experiments.

In the abnormal experiment with the fault source from stirring speed, the offline measured glutamate production showed that the fault started at 14 h, which was about 1.7 h later than the fault time shown by the proposed fault diagnosis approach (Fig. 4a). In the abnormal experiment with the fault source from the operation mistake that NaOH solution was used instead of ammonia water, the offline measured glutamate production showed that the fault started at 14 h, which was 1 h later than the fault time shown by the proposed approach (Fig. 5a). Further, unlike the proposed fault diagnosis approach, the fault diagnosis based on the offline measured glutamate production cannot diagnose the end of the fault when the fault source of fermentation conditions was rectified to normal. And thus, it is noteworthy that the online fault diagnosis based on the proposed approach was very simple and effective, compared with the fault diagnosis using offline measured glutamate production. The online fault diagnosis based on the combined approach of GAM and bootstrap identified not only the start of the fault in the fermentation process, but also the end of the fault when the fermentation contentions were rectified to normal. In addition, this approach only used the online recorded data on fermentation parameters for fault diagnosis during the fermentation process, without the requirement to measure the glutamate production by taking samples.

Our approach only included the significant factors that data can also be recorded online as the parameters in the fitted model for the online fault diagnosis, rather than a model including all factors that increase the complexity of the model for online fault diagnosis. But, the faults caused by the factors that were not parameters in the fitted model can be detected timely and effectively. For example, for the first abnormal fermentation with fault source from stirring speed, the fault was detected effectively by the estimated glutamate production that fell outside its 95 % confidence interval. In the second abnormal fermentation with the fault source from the human operation mistake, the factor pH was also not one of the parameters in the fitted GAM, but the fault was also detected timely and effectively by the fitted model. In addition, when NaOH solution was used instead of ammonia water, the level of pH was still maintained at a normal range during the operation mistake, but NaOH solution cannot serve as nitrogen source required by glutamate synthesis during the fermentation process as provided by the added ammonia water, and in this situation, the fault due to the lack of nitrogen source caused by the operation mistake was also revealed by the fitted model. These further indicate the effectiveness of the proposed approach for the online fault diagnosis in the fermentation process of glutamate.

## Conclusions

This study applied the GAM and bootstrap statistical methods for the first time to the online fault diagnosis in the fermentation process of glutamate with small samples. The fitted GAM using offline measured data on glutamate production and online recorded data on different fermentation parameters captured 99.6 % variance of glutamate production during fermentation process. The uncertainty of the estimated production of glutamate from the fitted GAM was quantified by bootstrap using 95 % confidence interval. The 95 % confidence interval for glutamate production were estimated from 1000 GAMs built by bootstrap re-sampling with replacement from the training data on glutamate production and fermentation parameters. The online fault diagnosis based on the proposed approach identified not only the start of the fault in the abnormal fermentation processes, but also the end of the fault when the fermentation conditions were back to normal. The proposed approach only need a small sample sets from normal fermentations experiments to establish the approach, and then use online recorded data on fermentation parameters for fault diagnosis in the fermentation process of glutamate, which was both time and cost-saving. Taking together, the proposed approach based on GAM and bootstrap provides a new and effective way for the online fault diagnosis in the fermentation process of glutamate with small sample sets.

## Methods

### Microorganism

The strain *C. glutamicum* S9114 used in this study was provided by the Key Laboratory of Industrial Biotechnology, Ministry of Education, Jiangnan University, China. Seed culture was grown in sterilized liquid medium consisting of the following components (in g/L): K_2_HPO_4_ 1.5, glucose 25, MnSO_4_ 0.005, FeSO_4_ 0.005, MgSO_4_ 0.6, corn slurry 25 and urea 2.5, with an initial pH of 7.0–7.2 on an Eberbach rotary shaker at 200 rpm and 32 °C for 8–10 h.

### Fermentation and data collection

The seed culture for glutamate production was then transferred into a 5 L fermenter (BIOTECH-5BG, Baoxing Co., China) with 3.4 L sterilized liquid medium consisting of the following components (in g/L): glucose 140, K_2_HPO_4_ 1.0, FeSO_4_ 0.002, MgSO_4_ 0.6, MnSO_4_ 0.002, thiamine 5.0 × 10^−5^, corn slurry 15 and urea 3.0, with an initial pH of 7.0–7.2 and at 32 °C. The pH was maintained at ~7.1 during the fermentation process by automatically addition of 25 % (w/w) ammonia water to the liquid medium. The added ammonia water also provided the nitrogen source required by glutamate synthesis during the fermentation process [24]. DO concentrations were controlled at different levels based on experimental requirements by automatically or manually controlled agitation speed. The CO_2_ and O_2_ concentrations in the inlet and exhaust gas under the partially pressure condition were measured online by a gas analyzer (LKM2000A, Lokas Co. Ltd., Korea). Glucose was added to the fermenter according to the requirement of substrate to ensure its concentration above a suitable level (15 g/L) during the fermentation process. The data on glutamate production were measured every 2 h and the data on different fermentation parameters (CER, DO, OUR, pH, SS and Temp) were online recorded every 6 min during the fermentation process. Data from five normal fermentation experiments were collected.

### Generalized additive model

Generalized additive model (GAM) is the generalization of linear models that estimate the relationship between response variable and smooth functions of explanatory variables in an additive form [27, 28, 44]. As an application of GAM, considering the continuous response variable $Y$ as the production of glutamate and explanatory variables $X_{1} , \ldots ,X_{p}$ as fermentation parameters (e.g., T, CER, DO, OUR, pH, SS, Temp), $Y$ is formulated as a sum of unspecified individual smooth functions of different fermentation parameters by an additive model:

2 $$Y = \,c + s(X_{1} ,m_{1} ) + s(X_{2} ,m_{2} ) + \cdots + s(X_{p} ,m_{p} ) + \varepsilon$$

where $$\varepsilon$$ is assumed to be normally distributed random errors with constant variance and a mean value of zero, and $$s(X_{i} ,m_{i} )$$$$(i = 1, \ldots ,p)$$ are smooth functions with efficient degree of freedom $$(m_{i} \ge 1)$$ to be estimated from data. Generalized linear model (GLM) is a special case of GAM when *m*_*i*_ = 1 [28]. GAM provides a useful extension of GLM where the smooth function $$s(X_{i} ,m_{i} )$$ gives the ability to examine the relationship between affected factor $$X_{i}$$ and the predicant *Y,* despite it is linearly or non-linear related.

To establish the model for the relationship between glutamate production and different fermentation parameters, data collected from normal fermentation experiments were used for constructing GLM and GAM as defined by Eq. (2). The offline data on glutamate production measured every 2 h and the online data on fermentation parameters (CER, DO, OUR, pH, SS and Temp) recorded every 6 min from five normal fermentation experiments were pooled together and then randomly separated into two groups referred to as the training data and testing data. The training data from four experiments were used to construct GLM and GAM, and the testing data from the remaining experiment were used to validate the fitted model. The best model is the one with highest value of adjusted *R*^2^, lowest generalized cross-validation (GCV) score and least significant components that can explain the effect of different fermentation parameters on glutamate production [28]. The performance of the fitted GAM was also measured based on the correlation coefficient and root mean square error between the estimated and measured production of glutamate from the testing data. The fitted GAM was used to estimate glutamate production during fermentation process using online recorded data of fermentation parameters T, CER, DO and OUR from five normal fermentation experiments.

### Bootstrap re-sample and confidence interval for glutamate production

To quantify the uncertainty of online estimated production of glutamate from the fitted GAM, a bootstrap method was then used to estimate the 95 % confidence interval for glutamate production. In general, a fitted GAM based on smoothing splines to the *N* groups sampling data $\{ (X_{i} (t),Y(t)):i = 1, \ldots ,p,t\,{ = }1, \ldots ,N\}$ is

3 $$\hat{Y}(t)\, = \,c + s(X_{1} (t),m_{1} ) + s(X_{2} (t),m_{2} ) + \cdots + s(X_{p} (t),m_{p} ) + \varepsilon$$

To quantify the uncertainty of glutamate production, a cumulative distribution function *G* for the confidence interval of the prediction error $$Y(N + h) - \hat{Y}(N + h)$$$$(h = 1, \ldots ,H)$$, with $$\hat{Y}(N + h) = c + \sum\nolimits_{k = 1}^{P} {s(X_{k} (N + h),m_{k} )}$$ using Eq. (3), was established. A $$100(1 - \alpha )\%$$ confidence interval for $$\hat{Y}(N + h)$$ based on $$X_{i} (N + h)_{{}}$$ was given as follow:

4 $$[\hat{Y}(N + h) + G^{ - 1} (\alpha /2),\hat{Y}(N + h) + G^{ - 1} (1 - \alpha /2)]$$

A bootstrap re-sampling approach [45, 46] was applied to estimate the confidence interval for $\hat{Y}(N + h)$ (glutamate production). The fitted GAM based on the training data was then used to calculate $\hat{Y}(t)$ and the residuals $e(t) = Y(t) - \hat{Y}(t)$. The error distribution *F* was estimated by the empirical distribution of residuals that were denoted $F_{n}$, and was then used to construct bootstrapped samples by the form:$\{ (X(t),Y^{*} (t)),t = 1,2, \ldots ,N\}$, $\{ (X(N + h),Y^{*} (N + h)),\,t = 1,2, \ldots ,H\}$ with $\hat{Y}^{*} (t) = \hat{Y}(t) + \varepsilon_{t}^{*}$ and $Y^{*} (N + h) = \hat{Y}(N + h) + \varepsilon_{N + h}^{*}$, where $\varepsilon_{t}^{*}$ and $\varepsilon_{N + h}^{*}$ were independently sampled from $F_{n}$; that was, they were randomly sampled with replacement from the set of residuals $\{ e_{1,} \ldots ,e_{N} \}$. The asterisk superscript denoted a value constructed for a particular bootstrap sample. Each bootstrapped sample was used to reconstruct GAM and get the estimated values $\hat{Y}^{*} (N + h)$, and the estimated errors $e_{N + h}^{'*} = Y^{*} (N + h) - \hat{Y}^{*} (N + h)$. The empirical distribution of $e_{N + h}^{'*}$, which was denoted $\tilde{G}$, was the estimated distribution of the bootstrap prediction errors, which can be used as the estimated distribution function *G* in Eq. (4). Therefore, $100(1 - \alpha )\%$ a confidence interval for can be $\hat{Y}(N + h)$ estimated as:

5 $$[\hat{Y}(N + h) + \tilde{G}^{ - 1} (\alpha /2),\hat{Y}(N + h) + \tilde{G}^{ - 1} (1 - \alpha /2)]$$

### Fault diagnosis

After obtaining the 95 % confidence interval for estimated glutamate production during the fermentation process, the proposed approach based on GAM and bootstrap was used for online fault diagnosis with a fault defined as an estimated production of glutamate fell outside the 95 % confidence interval. The fault diagnosis was conducted on two abnormal fermentation experiments of glutamate. The first experiment was with the fault source from abnormal stirring speed, and the other experiment was with the fault source from the human operation mistake that NaOH solution was used instead of ammonia water to maintain the pH level during the fermentation of glutamate.
